# Oral Health Care Reform in Finland – aiming to reduce inequity in care provision

**DOI:** 10.1186/1472-6831-8-3

**Published:** 2008-01-28

**Authors:** Teija Niiranen, Eeva Widström, Tapani Niskanen

**Affiliations:** 1 Päijät-Häme Joint Authority for Social and Health Services, Heinämaantie 9, 16300 Orimattila, Finland; 2 National Research and Development Centre for Welfare and Health, Stakes, P.O Box 220, 00530 Helsinki, Finland; 3 Institute of Clinical Dentistry, University of Tromso, 9037 Tromso, Norway

## Abstract

**Background:**

In Finland, dental services are provided by a public (PDS) and a private sector. In the past, children, young adults and special needs groups were entitled to care and treatment from the public dental services (PDS). A major reform in 2001 – 2002 opened the PDS and extended subsidies for private dental services to all adults. It aimed to increase equity by improving adults' access to oral health care and reducing cost barriers. The aim of this study was to assess the impacts of the reform on the utilization of publicly funded and private dental services, numbers and distribution of personnel and costs in 2000 and in 2004, before and after the oral health care reform. An evaluation was made of how the health political goals of the reform: integrating oral health care into general health care, improving adults' access to care and lowering cost barriers had been fulfilled during the study period.

**Methods:**

National registers were used as data sources for the study. Use of dental services, personnel resources and costs in 2000 (before the reform) and in 2004 (after the reform) were compared.

**Results:**

In 2000, when access to publicly subsidised dental services was restricted to those born in 1956 or later, every third adult used the PDS or subsidised private services. By 2004, when subsidies had been extended to the whole adult population, this increased to almost every second adult. The PDS reported having seen 118 076 more adult patients in 2004 than in 2000. The private sector had the same number of patients but 542 656 of them had not previously been entitled to partial reimbursement of fees.

The use of both public and subsidised private services increased most in big cities and urban municipalities where access to the PDS had been poor and the number of private practitioners was high. The PDS employed more dentists (6.5%) and the number of private practitioners fell by 6.9%. The total dental care expenditure (PDS plus private) increased by 21% during the study period. Private patients who had previously not been entitled to reimbursements seemed to gain most from the reform.

**Conclusion:**

The results of this study indicate that implementation of a substantial reform, that changes the traditionally defined tasks of the public and private sectors in an established oral health care provision system, proceeds slowly, is expensive and probably requires more stringent steering than was the case in Finland 2001 – 2004. However, the equity and fairness of the oral health care provision system improved and access to services and cost-sharing improved slightly.

## Background

**Figure 1:**
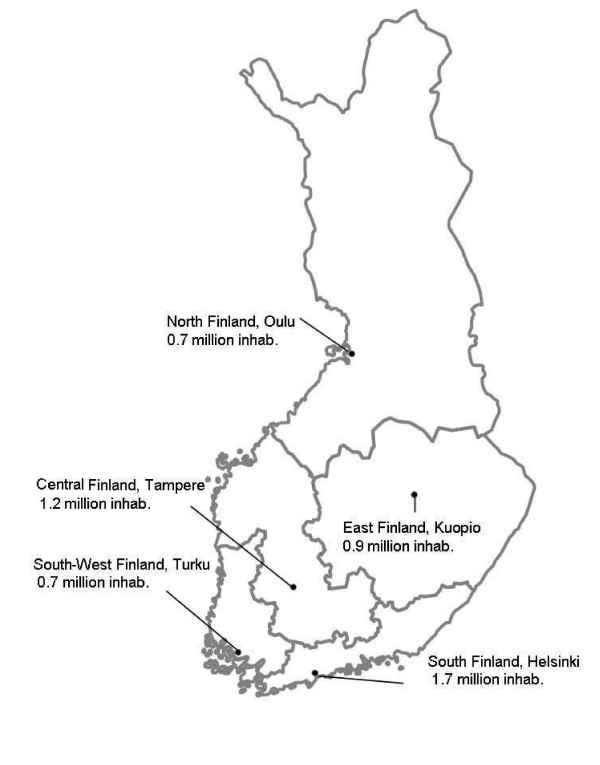
Geographical distribution of the population in Finland at 30 December 2004 into the University hospital regions of Helsinki, Turku, Tampere, Kuopio and Oulu. Total land area 338 000 km^2^.

Finland has two parallel systems for delivering dental care. They are the Public Dental Service (PDS) and private services. The PDS was established in 1972 to ensure the provision of dental services in sparsely populated areas (Figure 1). Since then it has been run at a local, rather than national, level by municipalities. In the PDS, patient fees are fixed and heavily subsidised. Public dental care for children and adolescents is free of charge. There is no control over prices charged for private dental services. However, since 1985 private fees can be partly subsidised by the national health insurance (NHI). The extent of such subsidy is limited as the NHI uses a fee schedule of its own when it subsidises a proportion of private fees for patients and fees in the NHI private fee scale are invariably less than those charged for private treatment. The PDS is financed by a combination of national and local taxation and patient's personal contributions to their fees. The NHI is financed by contributions from employers, employees and central funds (general taxation).

**Figure 2:**
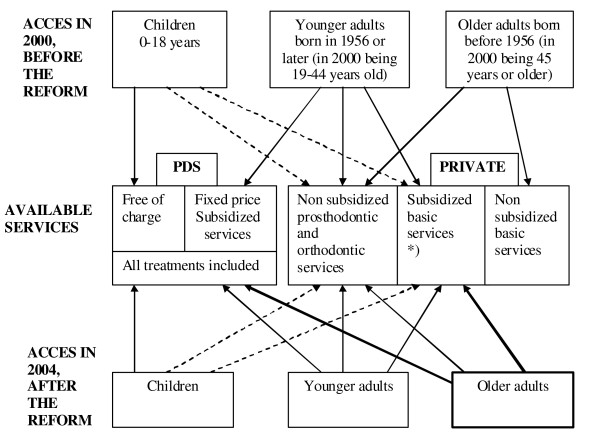
Access to Public Dental Services (PDS) and subsidized private services before and after the oral health care reform in Finland. *) subsidy of basic services in the private sector was about 45% in 2000 and 36% in 2004 of the costs depending on individual dentists' fees.

Following the introduction of the Primary Health Care Act in 1972, a slow expansion of the PDS began with the aim of including more adults, the pace of this change was limited by a lack of funds. In the 1980s remuneration of a proportion of the costs of private dental care by the NHI commenced (Table 1). Before the reform, in 2000, about a third of the Finnish population of 5.1 million lived in sparsely populated areas where the PDS offered dental treatment to all age groups. Another third lived in municipalities that offered dental care to those born in 1956 and later – as defined by the National Health Acts. The final third of the population lived in municipalities (usually big cities) that restricted dental care to children and young adults far below the recommended age limits (Table 1), basing their decisions on implicit or explicit local prioritisation. Thus, during the late 1990s, reports were published demonstrating relatively large differences, by age, place of residence and social background, of adults' use of dental care and in their perceived treatment needs. Much of the older adult population was virtually excluded from the publicly supported oral health care while the youngest and healthiest population received regular comprehensive care [1,2]. A clear inequality existed favouring the better off, especially in the use of private dental services [3]. In an attempt to improve the use of available resources and to meet the emerging oral health care needs and demands of the ageing population, a fundamental reform of the oral health care provision system was proposed. In 2000, the government changed the Primary Health Care Act and the National Health Insurance Law. This resulted, during 2001 – 2002, in the removal of the age limits restricting access to the PDS and changed the eligibility criteria to public subsidies for private care (Figure 2).

**Table 1:** Expansion of the Public Dental Service (PDS) and publicly subsidised private dental care in Finland 1970–2004 according to laws and regulations.

Year	Public Dental Service	Subsidised private services
1970–1979	Expansion in coverage* from 0–1 -year and 6–12 -year-olds to 0–18 -year-olds	Subsidised care when necessary for general health
1980–1989	Expansion to 19–31 -year-olds. Some special needs groups included:	Basic dental care** subsidised for 19–31 year-olds
	Pregnant women, students, seamen	
1990–1999	Expansion to 32–43 -year-olds. Some special needs groups included:	Expansion of the remunerations to 32–43 -year-olds. Some special needs groups included:
	Patients with radiation therapy to head and neck, World War II veterans	Patients with radiation therapy to head and neck, World War II veterans (prosthetic care also included)
2000–2004	The whole population without age limitations given access to the PDS in 2001–2002	Subsidised basic care for the whole population introduced in 2001–2002

* Inclusion of adult age groups into subsidized care has always been based on years of birth e.g. in 1980 adults born between 1961 and 1958 (18–31 years old) became eligible.

**Basic care includes all dental treatments except prosthetics and orthodontics.

The reform, aimed to improve all adults' oral heath by improving access to dental services. Another, in part political, aim was to increase equity in the use of services by reducing cost barriers. Services were to be offered based on medical or dental indications for treatment need and no longer on age or membership of a named special needs group, as had been the case previously. Thus the PDS was to offer dental care according to the same principles as those for primary medical care and the private services were to be seen as complementary – though important – to the PDS. The justification for the reform was the improvement in young people's oral health, that had taken place between 1980 and 2000 and greater demand for dental care by the increasingly dentate middle-aged and elderly who had put pressure for wider access to the PDS on politicians. Other factors that influenced the decision to reform included high numbers of dental professionals (relative to most other countries) and an improved national economy.

### Aims

Against this background, the aims of this study were to assess the impact of the oral health care reform, introduced in 2001–2002, on the utilization of publicly funded and private dental services, numbers of oral health care personnel and costs at both regional and national levels in 2000 and 2004, before and after the oral health care reform, using available data. The study also included an evaluation of how the health political goals of the reform: integrating oral health care into general health care, improving adults' access to care and lowering cost barriers had been fulfilled during the study period.

## Methods

In Finland, data for oral health care provided, oral healthcare workforce numbers and costs are reported at both municipal and national levels and then recorded in national registers [4-7], which were used as data sources for this study. The PDS register [4] collects data on the number of patients in three age groups (0–18 year old children and adolescents, adults born in 1956 and later, and older adults) reflecting the system for care and access to the PDS before the reform. Thus, in 2000 the adults who could access the PDS were 19–44 year olds and by 2004 they were the 18–48 year olds plus all older adults. Because the upper limit of free care was lowered in 2002 from 18 to 17 years, the youngest adult age group in 2000 was corrected accordingly to be comparable with the 2004 data. The NHI register [5] collects data on the number of National Health Insurance covered visits to private sector and NHI refunds.

The PDS and NHI data were classified according to the size and extent of urbanisation [8] and by geographical regions. The municipalities were classified into four groups:

• the ten biggest cities (population over 75 000),

• other urban municipalities (with at least 90% of population living in urban settlements or the population of the largest settlement with at least 15 000 inhabitants),

• semi-urban municipalities (with 60–90% of the population in urban settlements and the population of the largest settlement of 4 000 – 15 000 inhabitants)

• rural municipalities.

Geographically the data were divided into 5 groups corresponding with the University hospital regions (Figure 1). Data on dental care expenditure and funding [4,5] were converted to the price level of the year 2004 using the price index of public expenditure in Finland [9].

Comparisons were made between the PDS and private services in 2000 and in 2004, regarding numbers of patients, numbers of care providers [6,7] and changes in expenditure and funding. Differences between groups were compared statistically using the chi-square test or the t-test. A qualitative evaluation of the fulfilment of the health political goals of the reform was made on the basis of the results of this study and available national reports considering the study period.

## Results

### Use of dental services

In 2000, when access to publicly subsidised dental services for adults was restricted to those born in 1956 or later, every third adult had used the PDS or subsidised private services (Table 2) and four years later, in 2004, almost every second. The PDS reported 118 076 more adult patients than in 2000. In the private sector the total number of patients treated remained at the same level between 2000 and 2004. However, in 2004 of 1 008 630 privately treated patients, 542 656, who had not previously been eligible, received reimbursements.

**Table 2:** Numbers and proportions (%) of the adult population (over 17 years olds) who have used the Public Dental Services (PDS) or subsidised private dental services in Finland in 2000 and in 2004, before and after the Dental Care Reform.

**Year**	**Public Dental Services**		**Subsidised private services**		**All**	
	n	%	n	%	n	%
**2000**	846 138	21.5	465 446	11.8	1 311 584	33.3
**2004**	964 214	23.5	1 008 102	24.6	1 972 316	48.1

p-value		< 0,001		< 0,001		< 0,001

**Figure 3:**
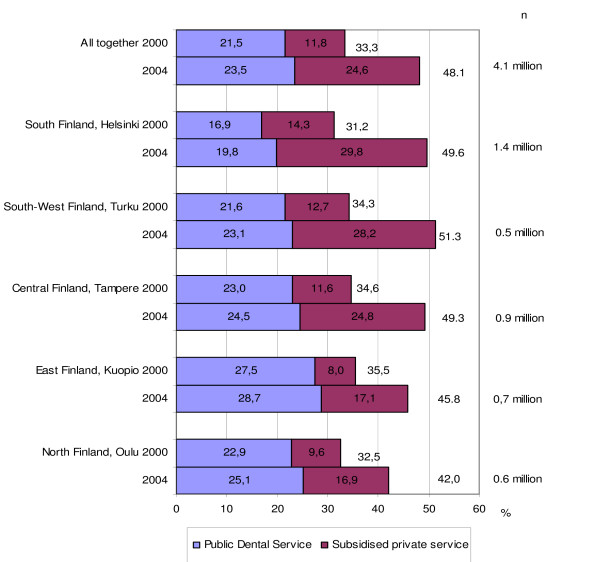
Proportions (%) of adults who used the Public Dental Service (PDS) or subsidised private services in Finland in 2000 and in 2004 by geographical regions (University hospital regions; n = number of adult inhabitants in the region). Pair wise comparisons (t-test) by region showed that the increase in use of both public (p < 0.01) and subsidized private dental services (p < 0.001) was statistically significant in all regions.

Before the reform adults' use of subsidised dental services (PDS + private) was highest in East Finland and lowest in South Finland (p < 0.001). After the reform, the use of services was significantly lower in the North (p < 0.001) than in the other regions (p < 0.001) (Figure 3). In all geographical regions the use of both public (p < 0.01) and subsidized private services (p < 0.001) increased significantly, 2000–2004 (Figure 3). The increase in the use of PDS was of about the same magnitude (1.2 – 2.9%-units) in all regions but the utilisation of subsidised private services increased more in the southernmost regions (15.5%-units) than in east and north (9.1 - 7.3%-units). In South and South West Finland adults used private services more than public services. The opposite was true in the North and East both before and after the reform.

The utilisation of the PDS increased most in big cities and other urban municipalities (2.6 – 2.9%-units) where previously use had been lowest (Table 3). The use of subsidised private services also increased most in densely populated municipalities, where it was already highest in 2000. The proportion of adult patients in the PDS increased from 48.5% to 53.4% (p < 0.001) and the proportion of children decreased from 51.5% to 46.6% (p < 0.001).

**Table 3:** Proportions (%) of the adult population who have used the Public Dental Services or subsidised private services in Finland in 2000 and 2004 by population density.

	Use of services (%)								
	PDS			Subsidised Private			All		
Municipalities grouped by population density in 2000	2000	2004	p-value	2000	2004	p-value	2000	2004	p-value

The ten biggest cities (n = 10)	14.3	17.2	< 0.001	15.5	30.0	< 0.001	29.8	47.2	< 0.001
Other urban municipalities (n = 56)	20.0	22.6	< 0.001	12.6	27.3	< 0.001	32.6	49.9	< 0.001
Semi urban municipalities (n = 75)	26.3	28.3	< 0.001	10.3	22.2	< 0.001	36.6	50.5	< 0.001
Rural municipalities (n = 291)	31.0	31.8	< 0.001	6.8	14.1	< 0.001	37.8	45.9	< 0.001

All (n = 432)	21.5	23.5	< 0.001	11.8	24.6	< 0.001	33.3	48.1	< 0.001

### Changes in staffing

The total number of working-age dentists (under 63 years) fell by 247 (5.3%) from 2000 to 2004. The PDS employed 131 dentists (6.5%) more and the private sector 143 dentists (6.9%) fewer after the reform. Population per dentist ratio increased from 1076 to 1146. The number of dental hygienists increased from 1022 to 1262 (23%) and population per dental hygienist ratio decreased from 5040 to 4122. No big changes occurred in the numbers of dental assistants and technicians.

The regional differences in the distribution of dentists did not change between 2000 and 2004. Thus in both 2002 and 2004, 66–68% of the public dentists and 80% of the private dentists worked in the three southernmost University hospital regions (Figure 1). The population per dentist ratio was higher in Central and East Finland than in other regions. The number of dental hygienists increased in all regions.

### Changes in expenditure and financing

The total dental care expenditure (PDS plus private services) increased by 21% from 2000 to 2004 (from EUR 584 million to EUR 708 million) (Table 4). The per-capita expenditure amounted to EUR 135 in 2004, which was 19% higher than that for 2000. The total running costs of the PDS, including the costs attributable to care of children and youngsters, increased by 25%. In the private sector, the cost increment was 18%. The greatest increase took place in expenditures on subsidised private care. The National Health Insurance (NHI) reimbursements increased by 106%, from EUR 46 million in 2000 to EUR 95 million, in 2004. The total cost per patient treated in 2004 in the PDS (EUR 186) was about 70% of that in the private sector (EUR 256) (Table 4).

**Table 4:** Dental care expenditure (EUR, %) by source of financing and per patients in the PDS and in the private services, 2000–2004, converted to 2004 prices using the price index of public expenditure.

	**PDS**				**Private**				**All**			
	**2000**		**2004**		**2000**		**2004**		**2000**		**2004**	
	EUR Million	%	EUR Million	%	EUR Million	%	EUR Million	%	EUR Million	%	EUR Million	%

**Financing**												
Paid by the patient	53	20	68	20	269	85	276	74	322	55	344	49
National Health Insurance	0		0		46	15	95	26	46	8	95	13
Local municipalities	216	80	269	80	0		0		216	37	269	38
**Expenditure**	**269**	100	**337**	100	**315**	100	**371**	100	**584**	100	**708**	100
												
Cost increment %				25				18				21
												
**Number of patients**	1 744 614^1^		1 807 161^2^		1 000 000^3^		1 028 630^4^		2 744 614		2 835 791	
												
**Cost per patient EUR**	154		186		218^5^		256^5^		167		212	
**Cost per inhabitant EUR**	52		64		60		71		112		135	

^1 ^The adult access to PDS was restricted. The number includes children. The amount of children was 898 476.

^2 ^The whole population was eligible for care. The number includes children. The amount of children was 842 947.

^3 ^Estimated numbers of all patients (who have had reimbursement and who have not had reimbursement). In 2000 the populations born in 1956 or later can have reimbursements

^4 ^Number of patients who have had reimbursements. In 2004 the whole populations can have reimbursements.

^5 ^Cost of reimbursed private care per patient (prosthetics and orthodontics not included)

In the PDS, the share of costs between patients (20%) and municipalities (80%) did not change. In the private sector the National Health Insurance financed a bigger part of the costs (in 2000, 15%; in 2004, 26%) and the patients' personal payments (out of pocket costs) decreased from 85% to 74% over the four year period.

Patients' payments were the largest source of oral health care funding before the reform in 2000, accounting for 55% of total cost of oral care. After the reform, in 2004, patients funded slightly less than half of the costs of all oral care (49%). Local authorities funded 37% of the costs in 2000 and 38% in 2004 through municipal tax revenues and the state support to the municipalities. The NHI funded the remaining balance of 8% in 2000 and 13% in 2004 (Table 4). About 40% of the NHI costs were covered by national taxes, 30% by the employers and 30% by the employees.

### Fulfilment of the reform goals

Table 5 lists the goals of the reform and summarizes the changes in the oral health care provision that had resulted by 2004. The intended integration of oral health care into the primary health care provision system in relation to equal access and care provided within a short time depending on need, proceeded smoothly. The reform highlighted the importance of oral health care as part of general health care and strengthened the position of the PDS. In addition, the PDS expanded without becoming dependent on profits from patients. The changes increased equity between citizens and lead to a fairer system.

**Table 5:** Evaluation of the fulfilment of the aims of the reform during the study period up to December 2004.

Main aims	Implementation	Evaluation of success
Integration of oral health care in the general health care provision system	- Access to the PDS became similar to that in the primary health care (the whole population) - Treatment according to need as in primary health care - Reimbursement of private care according to the same principles as in private primary health care	- Clear improvement in the principles of care provision in the PDS
Improved access to care for adults	- Minor increase in the number of patients seen in the PDS - Total number of private patients remained the same	- Minor improvements in access to the PDS, long queues for the PDS in a number of municipalities - No improvement in access to the private sector
Improved equity due to reduced cost barriers	- Subsidised treatments in the PDS opened for all adults - All private patients became eligible for reimbursements on an equal basis	- Society carried a bigger part of treatment costs as intended

Increasing access to the PDS and expanding the availability of partial reimbursement of private fees greatly increased adults' demand for dental services in both sectors [10,11] and consequently there were long waiting lists for care by the PDS, especially in a number of bigger cities which had previously heavily restricted adults' access to the PDS. In 2005, about 20% of the PDS clinics had not yet implemented the reform [12]. Failures to implement the reform were most marked in the largest cities, such as Helsinki, Turku and Tampere [13]. However, improvements were seen in the supply of emergency dental services in the PDS across the country [14,15]. Nevertheless, access to care did not improve as much as expected and especially in the private sector the average number of patients receiving partial reimbursement of fees who were treated per private sector dentist, remained low.

The 542 000 private patients who, prior to 2001, had not previously been eligible for partial reimbursement of private fees appear to have gained most from the reform. Even after the partial reimbursement from the NHI, private fees remained (and remain) higher than public fees. The results of a questionnaire study conducted in 2004, suggested that the increase in use of subsidized dental services was greatest in amongst those with a middle level of education [16]. Before the reform, the private sector was known to mainly cater for well-off adults interested in "investing in their teeth" [2,3,11]. It appears from our findings that the already well-off groups in urban areas in Southern Finland received greatest financial support for their oral health care. In addition, regional differences in the use of dental services increased. These latter findings suggest that the reform decreased inequities in oral care services, but created other new and different inequities.

## Discussion

Routine register data was used in this study. As dentists' remuneration in the PDS is partly based on these data and private patients' reimbursements by the NHI are fully based on recorded treatment, data on utilisation of dental services can be considered to be comprehensive in both sectors. One limitation was that because fewer than half of the adults treated in the private sector who were entitled to reimbursements only for reimbursable treatments, the total number of patients treated in the private sector had to be estimated. In 2000, fewer than half of the adults were entitled to such reimbursements whereas in 2004, all adults were entitled to reimbursements (except those who received a prosthetic care in the private sector). However, as few adults were likely to have received prosthesis without prior clinical examination or other treatments, which were eligible for partial reimbursement, a very small number would not have been recorded in the registers in 2004. A further potential complication in our estimates was that the PDS does not separate costs for the treatment of children and those for adults.

The oral health care reform was considered politically so important that the Parliament passed the changes more rapidly than proposed by the Ministry of Social Affairs and Health which supervises the health care provision system. In a period of two years (2001 to 2002) 2.1 million adults (40.9% of the population), who had previously not been eligible, became eligible for Public Dental Services or subsidised private care. During the four years covered in this study, there was little guidance for the PDS on how to proceed with the implementation of the reform. According to the chief dentists in the PDS, the main priorities were recruiting new dentists and hygienists and delegating to dental hygienists tasks previously done mainly by dentists [17]. However, the PDS had difficult recruiting dentists because in Finland the dental student intake was reduced in 1994 and two of the four undergraduate dental schools had been closed down. This and the large numbers of dentists reaching retirement age meant that the number of practising dentists has decreased in recent years. Furthermore, in the private sector the managers of larger dental companies complained of recruitment difficulties [11]. Although one dental school was reopened in 2000 and some recruitment has taken place from other European Union countries, there were many unfilled vacancies for dentists during the study period.

This study shows that there was a small increase in the supply of oral care services between 2000 and 2004. However, the mean number of patients seen in one year by a dentist in the PDS decreased from 863 to 840. On the other hand, the numbers of dental hygienists increased and they provided a greater proportion of children's treatments in the PDS than before [18]. In the private sector, the total number of patients remained at the same level as before the reform, but as the number of dentists decreased, the mean number of patients per year seen by a dentist increased from 480 (2000) to 525 (2004). The mean number of clinical working hours was similar in both the PDS and the private sector at about 30 hours per week in the PDS and about 28 hours per week in the private sector [19,20]. In Finland almost 70% of the dentists are women, which together with the freedom to set fees at any level in the private sector may in part explain the relatively low output in this care sector.

The chief dentists in the PDS complained that their dental staff did not support the speed of the reform. In particular, the changes required in work routines were often opposed locally [17]. There was no formal guidance for the private sector, which to a great extent continued to work as before. In the study period, in commercial terms, the inflation-adjusted growth of the private dental care industry revenue was high, and there was therefore no need to make any changes in marketing or pricing in spite of the fact that it now had to compete with the public sector [11].

A special survey conducted on treatments provided in the PDS in 2003 showed that a third of the adult patients had made emergency visits to a dentist [21]. This may have been because Parliamentary Ombudsman had taken a stand during the initial reform implementation and stated that emergency services were to be given maximum priority in the PDS in situations where it was not possible to offer care to all patients who sought care and treatment. Apart from this, in the initial phase of the reform, little change occurred in treatment provided by the PDS [18] or in the private sector [22].

The reform considerably increased the total running costs of oral health care. However, it should be noted that reducing costs and increasing efficiency were not primary goals of the reform. In 2004, the public sector saw 842 947 children and 964 214 adults (inclusive of the special needs groups) at a lower cost than the private sector which saw just over one million adults. The traditional distribution of the patients (*i.e*. the public sector catering for children, younger adults and special needs groups and the private sector for well-off middle-aged or older adults [2,3]), probably in part explains the differences in costs between the two sectors as the middle aged and older adults were more likely to receive treatment involving costs of crowns, bridges and dentures made at laboratories as well as clinical work. Overall, our study indicates that oral health care in the public sector (PDS) was less expensive than in the private sector.

The average rise in prices in the private sector between 2000 and 2004 was about 20% and the total NHI support for basic care provided by the private sector rose by 26% [5]. Thus, in practice, part of the cost of reimbursing private care and treatment was due to higher prices.

In most OECD countries, considerable inequities exist in general health care [23]. The Finnish oral health care reform aimed to reduce inequity and increase the fairness of the care provision system. The initial results showed some progress towards these goals. To speed up the reform process, in 2005, after the present study, the government introduced legislation to guarantee care in the PDS within "a reasonable period of time" but there have been no attempts to encourage the private sector to do more.

## Conclusion

The results of this study indicate that implementation of a substantial reform, that changes the traditionally defined tasks of the public and private sectors in an established oral health care provision system, proceeds slowly, is expensive and probably requires more stringent steering than was the case in Finland 2001 – 2004. However, the equity and fairness of the oral health care provision system improved and access to services and cost-sharing improved slightly.

## Competing interests

The author(s) declare that they have no competing interests.

## Authors' contributions

TeN: Principal investigator, collected data, performed statistical analyses, and wrote the manuscript.

EW: Main supervisor, designed the study, and wrote the manuscript.

TaN: Participated in the design of the study, collected data, and performed statistical analyses.

All authors read and approved the final manuscript.

## Pre-publication history

The pre-publication history for this paper can be accessed here:


